# The effect of single‐component sleep restriction therapy on depressive symptoms: A systematic review and meta‐analysis

**DOI:** 10.1111/jsr.14180

**Published:** 2024-02-28

**Authors:** Katrina Yan Kei Tse, Leonie Franziska Maurer, Colin Alexander Espie, Simon David Kyle

**Affiliations:** ^1^ Sir Jules Thorn Sleep and Circadian Neuroscience Institute, Nuffield Department of Clinical Neurosciences University of Oxford Oxford UK; ^2^ Mementor Leipzig Germany; ^3^ Big Health Inc. San Francisco California USA; ^4^ Big Health Inc. London UK

**Keywords:** cognitive behavioural therapy, depression, insomnia, meta‐analysis, psychological intervention, sleep restriction therapy

## Abstract

Sleep restriction therapy is a behavioural component within cognitive behavioural therapy for insomnia and is an effective standalone treatment for insomnia, but its effect on depressive symptoms remains unclear. This review aimed to synthesise and evaluate the impact of single‐component sleep restriction therapy on depressive symptoms relative to a control intervention. We searched electronic databases and sleep‐related journals for randomised controlled trials and uncontrolled clinical trials, published from 1 January 1986 until 19 August 2023, that delivered sleep restriction therapy to adults with insomnia. Random‐effects meta‐analysis of standardised mean differences and Cochrane risk of bias assessment were performed on randomised controlled trials, while uncontrolled clinical trials were discussed narratively. The meta‐analysis was pre‐registered on PROSPERO (ID: CRD42020191803). We identified seven randomised controlled trials (*N* = 1102) and two uncontrolled clinical trials (*N* = 22). Findings suggest that sleep restriction therapy is associated with a medium effect for improvement in depressive symptoms at post‐treatment (*N*_*c*_ = 6, *g* = −0.45 [95% confidence interval = −0.70 to −0.21], *p* < 0.001) and a small effect at follow‐up (*N*_*c*_ = 4, *g* = −0.31 [95% confidence interval = −0.45 to −0.16], *p* < 0.001). Five of the seven included randomised controlled trials were judged to have a high risk of bias. Standalone sleep restriction therapy appears to be efficacious for improving depressive symptoms at post‐treatment and follow‐up. However, conclusions are tentative due to the small number of trials and because none of the trials was performed in a population with clinically defined depression. Large‐scale trials are needed to test the effect of sleep restriction therapy in patients experiencing depression and insomnia. Findings also highlight the need to improve the standardisation and reporting of sleep restriction therapy procedures, and to design studies with more rigorous control arms to reduce potential bias.

## 1 INTRODUCTION

Insomnia disorder is a chronic sleep disorder characterised by difficulties initiating and/or maintaining night‐time sleep, which in turn causes distress and impairments during the day (American Psychiatric Association, 2013). Despite being under‐recognised and underdiagnosed (Grandner & Chakravorty, 2017; Grandner & Malhotra, 2015; Saleem et al., 2017), insomnia is highly prevalent, with about 10% experiencing symptoms severe enough to meet Diagnostic and Statistical Manual of Mental Disorder, Fifth Edition (DSM‐5) criteria for insomnia disorder (Davidson et al., 2019; Ree et al., 2017; Sateia et al., 2017; Wilson et al., 2019). Insomnia is associated with reduced quality of life (Kyle et al., 2010; LeBlanc et al., 2007) and confers marked risk for medical disorders (Sofi et al., 2014; Spiegelhalder et al., 2010) as well as mental disorders, such as major depressive disorder (Baglioni et al., 2011; Hertenstein et al., 2019).

Insomnia and depression are closely linked, and there is high comorbidity between the two (Staner, 2010). Individuals with insomnia are likely to experience more negative mood and less positive mood as a consequence of poor sleep (Buysse et al., 2007; Riedel & Lichstein, 2000). Additionally, sleep impairment is one of the key diagnostic criteria for depression (American Psychiatric Association, 2013). Of those individuals with a current depressive episode, more than 60% report having insomnia‐related sleep disturbances (Ohayon et al., 2000; Tsuno et al., 2005; Weissman et al., 1996; Yates et al., 2007). Furthermore, epidemiological studies reveal that insomnia is a key predictor for the onset of depression (Baglioni et al., 2011; Hertenstein et al., 2019) and increases the risk of depression twofold (Baglioni et al., 2011; Li et al., 2016). Complaints of disrupted sleep are one of the most common residual symptoms following depression remission (Carney et al., 2007), and confer an increased risk of relapse and recurrence (Baglioni et al., 2011; Li et al., 2016). Given the strong associations between insomnia and depression, treatments that alleviate both insomnia and depressive symptoms are important to provide optimal treatment outcome.

The recommended treatment of choice for insomnia disorder is cognitive behavioural therapy for insomnia (CBT‐I; Brasure et al., 2016; Qaseem et al., 2016; Riemann et al., 2023; Sateia et al., 2017; Schutte‐Rodin et al., 2008; World Health Organization, 2021). As a multicomponent psychological therapy, CBT‐I typically consists of sleep restriction therapy (SRT), stimulus control therapy, and cognitive therapies, supplemented by sleep hygiene education (SHE) and relaxation training (Baglioni et al., 2022; Morin & Benca, 2012). There is a large body of evidence demonstrating its efficacy in improving night‐time insomnia symptoms (Geiger‐Brown et al., 2015; Ho et al., 2015; Irwin et al., 2006; Koffel et al., 2015; Montgomery & Dennis, 2004; Murtagh & Greenwood, 1995; Okajima et al., 2010; Pallesen et al., 1998; Trauer et al., 2015), daytime insomnia symptoms (Benz et al., 2020) as well as depressive symptoms (Ballesio et al., 2018; Benz et al., 2020; Cunningham & Shapiro, 2018). In particular, multicomponent CBT‐I is associated with a medium effect (0.34–0.37) for improvement in depressive symptoms at post‐treatment (Ballesio et al., 2018; Benz et al., 2020). Furthermore, a recent review revealed that CBT‐I may be effective in preventing the development of depression in individuals without diagnoses of depression (Boland et al., 2023). These findings suggest sleep improvement may be a mechanism through which depressive symptoms improved and depression onset could be prevented.

Sleep restriction therapy (Spielman et al., 1987) is often considered the most active component of CBT‐I (Riemann et al., 2023), given its strong association with treatment outcomes (Maurer et al., 2021; Miller et al., 2014). It involves reducing excessive time in bed and reducing variability in bedtime and risetime, both of which are considered perpetuating factors in the maintenance of insomnia. According to the Triple R model (Maurer et al., 2018), the three key pathways through which SRT exerts its effect are: (1) restricting time spent in bed over successive nights directly strengthens homeostatic sleep drive and dampens pre‐sleep hyperarousal; (2) regularising sleep schedule tightens the circadian control of sleep and wakefulness, improving the stability and consolidation of sleep; (3) reconditioning the association between bedroom factors and sleep increases the likelihood that bedroom‐related stimuli will trigger sleep response.

Compared with multicomponent CBT‐I, single‐component SRT has a similar order of magnitude in improving self‐reported sleep continuity parameters and insomnia symptoms (Maurer et al., 2021; Okajima et al., 2010). In particular, Maurer et al.'s (2021) meta‐analysis revealed that SRT has a medium‐to‐large effect size for improving self‐reported sleep continuity parameters (i.e. sleep‐onset latency, wake‐time after sleep onset and sleep efficiency [SE]) and a large effect size for improving insomnia severity (measured by the Insomnia Severity Index) compared with control at post‐treatment. Furthermore, the American Academy of Sleep Medicine (AASM) clinical practice guidelines in 2021 (Edinger et al., 2021) gave a conditional recommendation in favour of SRT as treatment of chronic insomnia in adults. As a protocol‐based, single‐component treatment, SRT may have the potential to be integrated with depression treatment to improve sleep outcomes in patients experiencing depression. However, it is currently unclear to what extent SRT is effective in improving depressive symptoms. Understanding the effect of SRT on depressive symptoms could also offer valuable insights into the role of sleep regularity and sleep continuity in improving depression.

### 1.1 Objectives

Previous meta‐analyses have primarily focused on the effects of multi‐component CBT‐I on depressive symptoms, but little is known about the effectiveness of SRT on depressive symptoms. Thus, the current study aimed to appraise the evidence for single‐component SRT on depressive symptoms in individuals with insomnia relative to a control intervention.

## 2 METHODS

The systematic review and meta‐analysis were undertaken in compliance with PRISMA guidelines for the reporting of systematic reviews (Page et al., 2021). We included multiple study designs in our search, including randomised controlled trials (RCTs) and uncontrolled clinical trials (UCTs), to provide a comprehensive overview of the literature available. Meta‐analysis was conducted on RCTs, while UCTs were discussed narratively. The protocol for the meta‐analysis was pre‐registered on PROSPERO (ID: CRD42020191803). We initially planned, as a secondary objective, to also extract and analyse mood‐related outcomes beyond depression; however, our search only yielded one study, precluding any meaningful syntheses. We do however include a table describing this study in the supplement (Appendix 5).

### 2.1 Search strategy

Published and unpublished studies were identified by searching electronic databases (PubMed, Web of Knowledge, Scopus, CENTRAL, and PsychINFO), clinical trial registries (ClinicalTrials.gov, ISRCTN Registry and WHO International Clinical Trials Registry Platform) and journals focused on sleep research (*Journal of Sleep Research*, *Sleep*, *Sleep Medicine*, *Behavioural Sleep Medicine*). The database coverage included studies published in English between 1 January 1986 (1 year before the publication of the SRT guidelines by Spielman et al., 1987) and 19 August 2023. The search strategy used for all database platforms was the same as the previous meta‐analyses of SRT (Maurer et al., 2018; Miller et al., 2014): “insomnia”, “sleep disorder” or “sleep disturbance”, AND “sleep restrict*”, “sleep compress*”, “bedtime restriction” or “time in bed restriction” in title, abstract or keyword (see Appendix 6 for full line by line search strategy in each database). Additionally, reference lists of the included studies were searched for potentially relevant studies.

All citations produced by the search strategy were exported to a reference management software (Mendeley, Elsevier, London, UK) where duplicates were removed. Search results were screened for relevance based on title and abstract by the first author (KT). Records that did not include individuals with insomnia and did not implement SRT were excluded based on title and/or abstract. Full‐text articles were then obtained for the screened citations, which were subsequently screened further by KT using the eligibility criteria below. Study selection was discussed between authors (KT, LM and SK) where there was uncertainty until consensus was reached.

### 2.2 Eligibility criteria

Inclusion criteria were as follows. (1) Adults (≥ 18 years old). (2) Sample fulfils the criteria for insomnia disorder, as verified by diagnostic criteria; or self‐report symptoms of insomnia, assessed by validated questionnaires, clinical interviews or sleep diary criteria. Individuals with insomnia comorbid with other medical or mental health conditions were included as long as the focus was on insomnia. (3) Implemented a standalone SRT intervention (or in combination with sleep hygiene). Studies that delivered a modification of SRT were included as long as the treatment involved systematic reduction of time in bed for the treatment of insomnia, consistent with the approach of Spielman et al. (1987), whereby an initial, curtailed sleep window was prescribed with the aim of extending over subsequent weeks based on pre‐specified SE criteria. (4) Outcomes included mean scores of depressive symptoms using validated depression questionnaires. (5) RCTs and UCTs published in English between 1986 and 2023. (6) For RCTS, permitted control conditions included receiving no treatment, wait list control (WLC), treatment as usual, or control interventions that have no evidence for improving sleep or insomnia (i.e. sleep hygiene; Edinger et al., 2021).

Exclusion criteria were: (1) conference abstract, dissertation, letter, case study or review; (2) applied sleep compression as the intervention, which involved using a more incremental and progressive approach to decreasing time in bed; or (3) implemented SRT as a package treatment (e.g. brief behavioural intervention for insomnia, which combines SRT with stimulus control).

### 2.3 Data extraction

The following characteristics were extracted from the RCTs and UCTs: (1) publication year; (2) geographic location; (3) study design; (4) total number of participants randomised, and the number of female participants; (5) mean age and standard deviation; (6) method of recruitment; (7) hypnotic or psychiatric medication status; (8) comorbidity; (9) diagnostic criteria for insomnia; (10) intervention format; (11) control group; (12) measure of depressive symptoms. We also extracted information on SRT characteristics for each study: (1) sleep window generation; (2) minimum time in bed; (3) SE criteria and change to sleep window; (4) position of the sleep window. The first author (KT) extracted the study characteristics.

Two researchers (KT, LM) independently extracted summary data from RCTs (means and standard deviations of depressive symptoms, and number of participants providing outcome data) from each intervention group (SRT and control) at all available timepoints, and cross‐checked their findings. Where there was uncertainty, the authors (KT, LM and SK) discussed until consensus was reached and reached out to corresponding authors where necessary.

### 2.4 Risk of bias in individual studies

All RCTs were appraised using the Revised Cochrane risk‐of‐bias tool for randomised trials (RoB2; Sterne et al., 2019). Studies were rated against the following criteria: (1) randomisation process; (2) deviations from intended interventions; (3) missing outcome data; (4) measurement of the outcome; (5) selection of the reported result. The overall rating varies between low risk, some concerns, or high risk. Two researchers (KT, LM) independently evaluated the risk of bias in each study and cross‐checked their findings. Where there was a discrepancy in the appraisal of the risk of bias, the authors (KT, LM, SK) discussed until a consensus was reached.

### 2.5 Data synthesis and analysis

The primary outcome for this review was self‐reported depressive symptoms at post‐treatment for RCTs. Exploratory analysis was conducted on self‐reported measures of depressive symptoms at follow‐up. Effect sizes and 95% confidence intervals (CIs) were calculated for the between‐group differences at post‐treatment and follow‐up. Post‐treatment was defined as the assessment point after treatment completion; follow‐up was defined as the single next assessment point after post‐treatment.

Since studies assessed depressive symptoms using different psychometric scales, standardised mean difference was used to standardise the findings of the studies to a uniform scale before they were combined. This was conducted by calculating the size of the treatment effect (difference in mean outcome between groups) relative to the variability of the study (standard deviation of outcome among participants).

Hedges' *g* (Hedges, 1981) was the effect size statistic for this meta‐analysis. Hedges' *g* was expressed as the difference between the mean of the treatment group against the mean of the control group, relative to the pooled weighted standard deviation:

$$g=\frac{\mu_{\mathrm{tx}}-\mu_{\text{ctrl}}}{s},$$

where $\mu_{\mathrm{tx}}$ and $\mu_{\text{ctrl}}$ denote the mean of the treatment group and control group, respectively, and *s* denotes the pooled weighted standard deviation (SD):

$$s=\frac{\sqrt{\left(n_{\mathrm{tx}}-1\right){\mathrm{SD}}_{\mathrm{tx}}^{2}+\left(n_{\text{ctrl}}-1\right){\mathrm{SD}}_{\text{ctrl}}^{2}}}{n_{\mathrm{tx}}+n_{\text{ctrl}}-2},$$

where $n_{\mathrm{tx}}$ and $n_{\text{ctrl}}$ denote the number of participants in treatment and control groups, respectively, and ${\mathrm{SD}}_{\mathrm{tx}}$ and ${\mathrm{SD}}_{\text{ctrl}}$ denote the standard deviation of treatment and control groups, respectively. Effect sizes were interpreted as either small (0–0.32), medium (0.33–0.55) or large (≥ 0.56; Lipsey & Wilson, 1993). Effect sizes were calculated using published means and standard deviation. Where studies reported other measures of dispersion, such as standard errors (SE), the following formula was used to transform to standard deviation:

$$\mathrm{SD}=\mathrm{SE}\times \sqrt{N},$$

where *N* denotes the sample size.

Meta‐analyses were performed using the meta 6.2‐1 package (Balduzzi et al., 2019) in R (version 4.2.2). The inverse‐variance method was used to conduct the meta‐analysis. The random effects model was employed as we expected heterogeneity between the studies (e.g. different SRT components, different control groups). The Wald‐type CI was presented. Restricted maximum likelihood estimator was used to estimate between‐study variance (Veroniki et al., 2016). Heterogeneity was inspected using *I* ^2^ statistics, which describes the percentage of variation across studies (Higgins et al., 2003; Higgins & Thompson, 2002). We defined *I* ^2^ values as either low (0%–30%), moderate (31%–50%), substantial (51%–75%) or considerable heterogeneity (76%–100%). The 95% CI around *I* ^2^ were calculated. In line with the recommendations from the Cochrane Handbook for Systematic Reviews of Interventions (Higgins & Green, 2022), for meta‐analysis with less than 10 studies, publication bias was not assessed. Forest plots were created to provide graphical overview of the meta‐analysis.

## 3 RESULTS

### 3.1 Study selection

**FIGURE 1:**
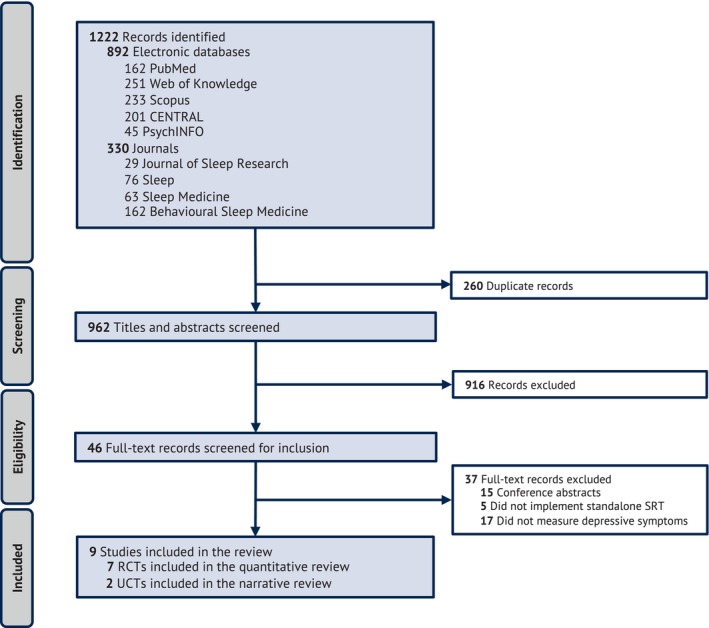
PRISMA flowchart showing the process of study selection of the systematic review. RCT, randomised controlled trials; SRT, sleep restriction therapy; UCT, uncontrolled clinical trials.

We identified 1222 records from the search. After removing duplicates, we screened 962 titles and abstracts, of which 916 records were excluded for not delivering SRT as a standalone intervention, not including measures of depressive symptoms, or not having exclusively adults as participants. We then assessed 46 full‐text studies for eligibility, of which 37 studies were excluded because: (1) it was a conference abstract (*n* = 15); (2) standalone SRT was not implemented (*n* = 5); and (3) measures of depressive symptoms were not included (*n* = 17). The list of excluded studies from full‐text screening can be found in Table S6 in Appendix 7. In total, nine studies were eligible for inclusion in the review, of which seven studies were RCTs and were included in the meta‐analysis, while two studies were UCTs and were discussed narratively (Figure 1). One study (Falloon et al., 2015) allowed participants to be as young as 16 years to participate. We nevertheless included this study because their baseline characteristics showed that participants were clearly from the adult population (mean age, SRT = 55.4 ± 12.7; control = 51.8 ± 13.4).

### 3.2 Study characteristics

Table 1 summarises the characteristics of studies included in the review. The earliest study was published in 2012 (Epstein et al., 2012), and the latest in 2023 (Kyle et al., 2023). RCTs recruited more participants (*n* range: 49–642) than UCTs (*n* range: 7–15). The number of participants from Kyle et al. (2023) represented 62% of the total number of participants in the meta‐analysis at post‐treatment (total *N*_p_ = 847, Kyle *N*_p_ = 528) and 70% at follow‐up (total *N*_p_ = 735, Kyle *N*_p_ = 512). Three studies recruited participants from the community (Epstein et al., 2012; Lancee et al., 2019; Maurer et al., 2020), two studies recruited participants from primary care (Falloon et al., 2015; Kyle et al., 2023), and four studies recruited from both community and primary care settings (Aji et al., 2020; Gieselmann & Pietrowsky, 2019; Kalmbach et al., 2019; Krieger et al., 2019). Six studies recruited participants who were free of medication (Aji et al., 2020; Epstein et al., 2012; Falloon et al., 2015; Kalmbach et al., 2019; Lancee et al., 2019; Maurer et al., 2020), and six studies excluded psychiatric comorbidity (Aji et al., 2020; Epstein et al., 2012; Falloon et al., 2015; Kalmbach et al., 2019; Krieger et al., 2019; Maurer et al., 2020). Depression severity was not a primary outcome in any of the included studies, and none of the studies specifically recruited patients with a diagnosis for depression (see Appendix 2 for baseline score of depression measure); though Lancee 2019 was the only included study to have depression as part of their inclusion criteria. Five studies (Falloon et al., 2015; Gieselmann & Pietrowsky, 2019; Kalmbach et al., 2019; Krieger et al., 2019; Maurer et al., 2020) recruited participants with depression scores classified as “normal”/“mild depression”, and two studies (Epstein et al., 2012; Kyle et al., 2023) recruited participants with depression scores classified as “moderate depression” (Appendix 2). In all studies, the diagnostic criteria for insomnia were verified using validated questionnaires, clinical interviews and/or sleep diary. Hypnotic medication was allowed in three studies (Gieselmann & Pietrowsky, 2019; Krieger et al., 2019; Kyle et al., 2023) and psychiatric comorbidity was allowed in three studies (Gieselmann & Pietrowsky, 2019; Kyle et al., 2023; Lancee et al., 2019).

**TABLE 1:** Characteristics of studies included in the review.

Author, year, *location*	Study design	Sample size randomised (female), mean age (SD), recruitment	Hypnotic medication status, comorbidity	Diagnostic criteria of insomnia (*determined by*)	Intervention & its format	Control	Depression measure (PT, FU)	Risk of bias
Kyle et al. (2023), *UK*	RCT	*N* = 642 (489 f) SRT (*n* = 321): 55.7 years (15.3) SHE (*n* = 321): 55.2 years (16.5) Adults from medical practices	Medication allowed, psychiatry comorbidity allowed	DSM‐5 Insomnia Disorder (*SCI*)	4‐session SRT Individual, FtF (S1, S3), Phone (S2, S4)	SHE	PHQ‐9 (3 m, 6 m, 12 m)	Some risk of bias
Maurer et al. (2020), *UK*	RCT	*N* = 56 (39 f) SRT (*n* = 27): 40.63 years (9.13) TBR (*n* = 29): 40.93 years (9.24) Adults from community	Medication free, psychiatric comorbidity excluded	DSM‐5 Insomnia Disorder (*SCI ≤ 16 and telephone interview*)	4‐week SRT Individual, FtF (1, 2 week), Phone (3, 4 week)	TBR	HADS‐D (4 wk, 3 m)	Some risk of bias
Krieger et al. (2019), *Switzerland*	RCT	*N* = 62 (45 f) SRT (*n* = 41): 46.59 years (17.52) CAU (*n* = 21): 45.24 years (12.40) Adults from physician referrals and community	Medication allowed, psychiatric comorbidity excluded	ICSD‐3 Insomnia Disorder (*Telephone interview*)	8‐week SRT Internet‐based, one session per week	CAU	ADS‐K (8 week, 6 m)	High risk of bias
Kalmbach (2019), *USA*	RCT	*N* = 102 (102 f) SRT (*n* = 52): 56.65 years (4.95) SHE (*n* = 50): 57.34 years (5.97) Postmenopausal women from primary care, sleep clinic and community	Medication free, depression comorbidity excluded	DSM‐5 Insomnia Disorder Self‐reported WASO of ≥ 1 hr on ≥ 3 nights per week; objective WASO of ≥ 45 min (*Clinical interview & 2 nights of PSG*)	2‐week SRT Individual, FtF (initial & ending session), phone (every 3–4 days between 1 and 2 weeks)	SHE	BDI‐II (4 week, 6 m)	High risk of bias
Gieselmann (2019), *Germany*	RCT	*N* = 49 (25 f) SRT (*n* = 27): 39.3 years (14.47) WLC (*n* = 22): 42.74 years (11.73) Adults from medical practice, university and community	Medication allowed, psychiatric comorbidity allowed	Insomnia disorder SOL of > 30 min for ≥ 3 nights per week over the last 6 months (*Clinical interview*)	3‐week SRT (combined with imagination exercise and sleep hygiene advise), individual, FtF (1, 3 weeks)	WLC	CES‐D (4 week, 2 m)	High risk of bias
Falloon (2015), *New Zealand*	RCT	*N* = 97 (75 f) SSR (*n* = 46): 55.4 years (12.7) SHE (*n* = 51): 51.8 years (13.4) Adults from general practices	Medication free, psychiatric comorbidity excluded	Insomnia disorder for ≥ 6 months Sleep difficulties on ≥ 3 nights per week (*Questionnaires, physical examination, sleep diary at baseline*)	3‐week SSR Individual, FtF (1, 3 weeks)	SHE	PHQ‐9 (/, 6 m)	High risk of bias
Epstein (2012), *Canada*	RCT	*N* = 94 (57 f) SRT (*n* = 44): 68.00 years (8.25) WLC *(n* = 50): 69.50 years (8.34) Older adults from community	Medication free, psychiatric comorbidity excluded	Insomnia Disorder for ≥ 6 months sleep onset or maintenance of ≥ 45 min per night for ≥ 3 nights per week; Complained of impaired daytime functioning (*2‐week sleep diary at baseline*)	6‐week SRT (with sleep hygiene instructions), Group (4–6 individuals), FtF (1, 2, 4 weeks), Phone (5–6 weeks)	WLC	GDS (6 week, /)	High risk of bias
Aji (2020), *Australia*	UCT	*N* = 15 (15 f) 40.0 years (14.2) Adults from clinician referrals and community	Medication free, severe psychiatric comorbidity excluded	DSM‐5 Insomnia Disorder (*ISI ≥ 15, PSQI > 5, clinical interview*)	3‐week SRT Mobile app	/	HADS‐D (6 week, /)	/
Lancee (2019), *Netherlands*	UCT	*N* = 7 (4 f) 45.7 years (range 28–69) Adults from community	Medication free, insomnia with depressive symptoms, psychiatric comorbidity allowed	Insomnia disorder (*SCID‐5‐RV, ISI ≥ 10, SE ≤ 85, clinical interview*)	6‐week SRT Individual, FtF (1, 3 weeks), phone (2, 4, 5, 6 weeks)	/	PHQ‐9 & BDI‐II (6 week, 3 & 6 m)	/

Abbreviations: ADS‐K, Allgemeine Depressions‐Skala – Kurzform (German version of CES‐D); BDI‐II, Beck Depression Inventory II; CAU, care as usual; CES‐D, Center for Epidemiological Studies Depression Scale; DSM‐5, Diagnostic and Statistical Manual of Mental Disorder, Fifth Edition; FtF, face to face; FU, follow‐up; GDS, Geriatric Depression Scale; HADS‐D, Hospital Anxiety and Depression Scale – Depression Subscale; ICSD‐3, International Classification of Sleep Disorders, Third Edition; ISI, Insomnia Severity Index; PHQ‐9‐D, Patient Health Questionnaire – Depression Scale; PSG, polysomnography; PSQI, Pittsburgh Sleep Quality Index; PT, post‐treatment; RCT, randomised controlled trial; SCI, sleep condition indicator; SCID‐RV, Structured Clinical Interview for DSM‐5 – Research Version; SE, sleep efficiency; SHE, sleep hygiene education; SOL, sleep‐onset latency; SRT, sleep restriction therapy; SSR, simplified sleep restriction; TBR, time in bed regularisation; UCT, uncontrolled clinical trial; WASO, wake after sleep onset; WLC, wait list control; S, session; m, month.

Of the nine studies, eight (Aji et al., 2020; Epstein et al., 2012; Gieselmann & Pietrowsky, 2019; Kalmbach et al., 2019; Krieger et al., 2019; Kyle et al., 2023; Lancee et al., 2019; Maurer et al., 2020) delivered SRT, while one (Falloon et al., 2015) delivered simplified sleep restriction (SSR), a modified version of SRT where initial sleep window is generated from self‐reported total sleep time plus 50% of the time spent awake in bed. We have created a breakdown of SRT procedures from included studies using guidelines as recommended by Kyle et al. (2015; Appendix 1). Four studies (Epstein et al., 2012; Falloon et al., 2015; Kyle et al., 2023; Maurer et al., 2020) provided information on all SRT parameters. All studies that reported the information set the sleep window based on diary‐reported total sleep time and positioned the sleep window based on patient preference. There was variability in minimum sleep window time, SE criteria and sleep window change. The duration of the SRT intervention ranged from 2 to 8 weeks. Six studies delivered individual intervention (Falloon et al., 2015; Gieselmann & Pietrowsky, 2019; Kalmbach et al., 2019; Kyle et al., 2023; Lancee et al., 2019; Maurer et al., 2020), one delivered group intervention (Epstein et al., 2012) and two delivered the intervention digitally (Aji et al., 2020; Krieger et al., 2019). Five studies delivered the intervention using both face‐to‐face sessions and phone calls (Epstein et al., 2012; Kalmbach et al., 2019; Kyle et al., 2023; Lancee et al., 2019; Maurer et al., 2020), while two used face‐to‐face sessions only (Falloon et al., 2015; Gieselmann & Pietrowsky, 2019). As for the control group, three RCTs used passive control groups, including WLC (Epstein et al., 2012; Gieselmann & Pietrowsky, 2019) and care as usual (CAU; Krieger et al., 2019), while three studies used SHE as minimally active control (Falloon et al., 2015; Kalmbach et al., 2019; Kyle et al., 2023) and one study used time in bed regularisation (TBR) as an active control (Maurer et al., 2020). The treatment rationale of TBR was bedtime consistency/regularity of bedtime and rise time, which is a common theme within SHE.

Measures of depressive symptoms were self‐reported questionnaires that assessed state‐level symptoms within a specific time frame, these included Beck Depression Inventory II (BDI‐II; Beck et al., 1996), Center for Epidemiological Studies Depression Scale (CES‐D; Radloff, 1977), Patient Health Questionnaire (PHQ‐9; Kroenke et al., 2001), Hospital Anxiety and Depression Scale (HADS; Zigmond & Snaith, 1983) and the Geriatric Depression Scale (GDS; Yesavage et al., 1982). The post‐treatment period was between 4 and 12 weeks, while the follow‐up period was between 2 and 6 months. Kyle et al. (2023) also had a 12‐month follow‐up assessment in addition to their 6‐month follow‐up. Only the 3‐month post‐treatment and 6‐month follow‐up from Kyle et al. (2023) were included in the meta‐analysis given it was closest to timepoints of other RCTs.

### 3.3 Risk of bias within RCT studies

**FIGURE 2:**
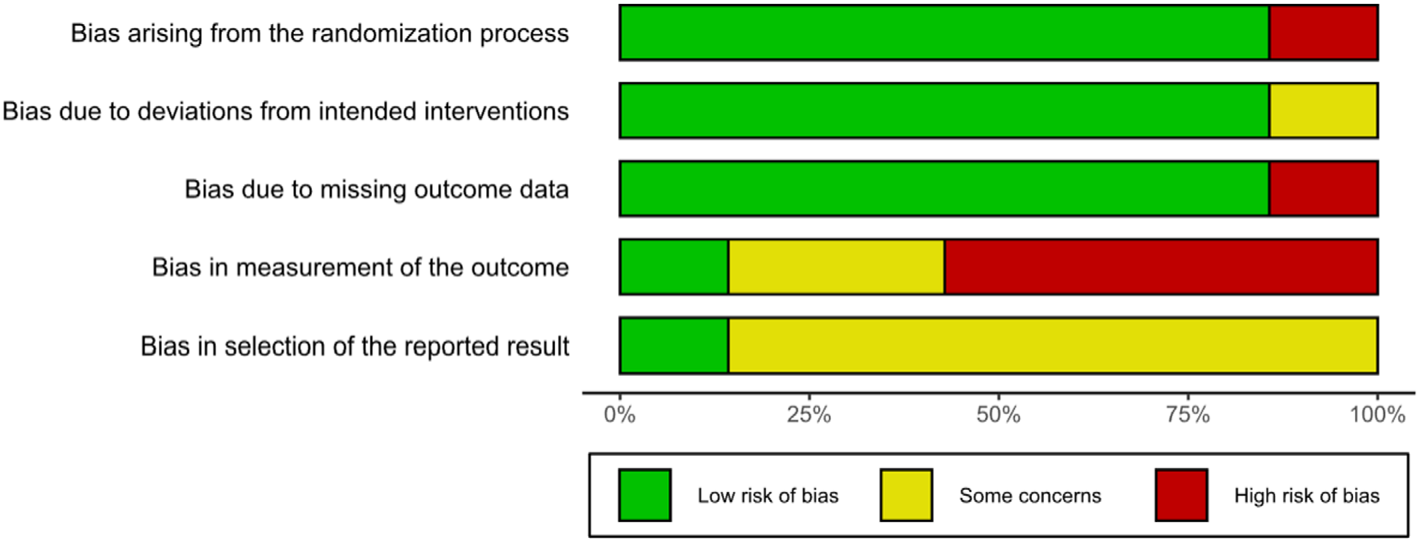
Risk of bias summary.

Figure 2 presents the quality assessment of the included RCT studies. Overall, we judged five studies (Epstein et al., 2012; Falloon et al., 2015; Gieselmann & Pietrowsky, 2019; Kalmbach et al., 2019; Krieger et al., 2019) to have a high risk of bias, and we raised some concerns of bias for two studies (Kyle et al., 2023; Maurer et al., 2020). All studies had an adequate randomisation procedure. Six studies (Epstein et al., 2012; Falloon et al., 2015; Kalmbach et al., 2019; Krieger et al., 2019; Kyle et al., 2023; Maurer et al., 2020) had an adequate allocation concealment, whereas one (Gieselmann & Pietrowsky, 2019) did not. Six studies (Epstein et al., 2012; Falloon et al., 2015; Gieselmann & Pietrowsky, 2019; Krieger et al., 2019; Kyle et al., 2023; Maurer et al., 2020) used treatment protocols to avoid deviations from intended interventions and used intention to treat analysis, while one (Kalmbach et al., 2019) did not. Six studies (Epstein et al., 2012; Falloon et al., 2015; Gieselmann & Pietrowsky, 2019; Krieger et al., 2019; Kyle et al., 2023; Maurer et al., 2020) reported completeness of outcome data and provided details on attrition rate, while one study (Kalmbach et al., 2019) did not.

Being aware of the assigned intervention could create bias when measuring self‐reported depressive symptoms, which were assessed using participant‐reported questionnaires in all studies. Three studies (Epstein et al., 2012; Gieselmann & Pietrowsky, 2019; Krieger et al., 2019) used WLC or CAU as a minimally active control arm, which did not require any engagement with the treatment, making knowledge of the assigned treatment likely to bias the outcome assessment. Two studies (Kalmbach et al., 2019; Kyle et al., 2023) delivered SHE as a minimally active control arm through weekly emails (Kalmbach et al., 2019), a booklet (Kyle et al., 2023) or through verbal advice from the study general practitioner (Falloon et al., 2015). Although SHE did not match for time with the therapist and treatment structure with the intervention group (i.e. regular sessions), it required active engagement with the treatment (i.e. following SHE advice), making knowledge of the assigned treatment less likely to bias the outcome assessment. The control treatment from Maurer et al. (2020), TBR, matched well with SRT for time with the therapist, treatment engagement, and treatment structure, so knowledge of the assigned treatment was less likely to affect outcome assessment. Finally, only one study (Kyle et al., 2023) was pre‐registered, five studies (Epstein et al., 2012; Gieselmann & Pietrowsky, 2019; Kalmbach et al., 2019; Krieger et al., 2019; Maurer et al., 2020) registered the aims of the trial retrospectively, while one study (Falloon et al., 2015) had no registration for the trial. Appendix 3 presents the breakdown of each RoB2 item for each study.

## 4 META‐ANALYSIS

### 4.1 At post‐treatment

**FIGURE 3:**
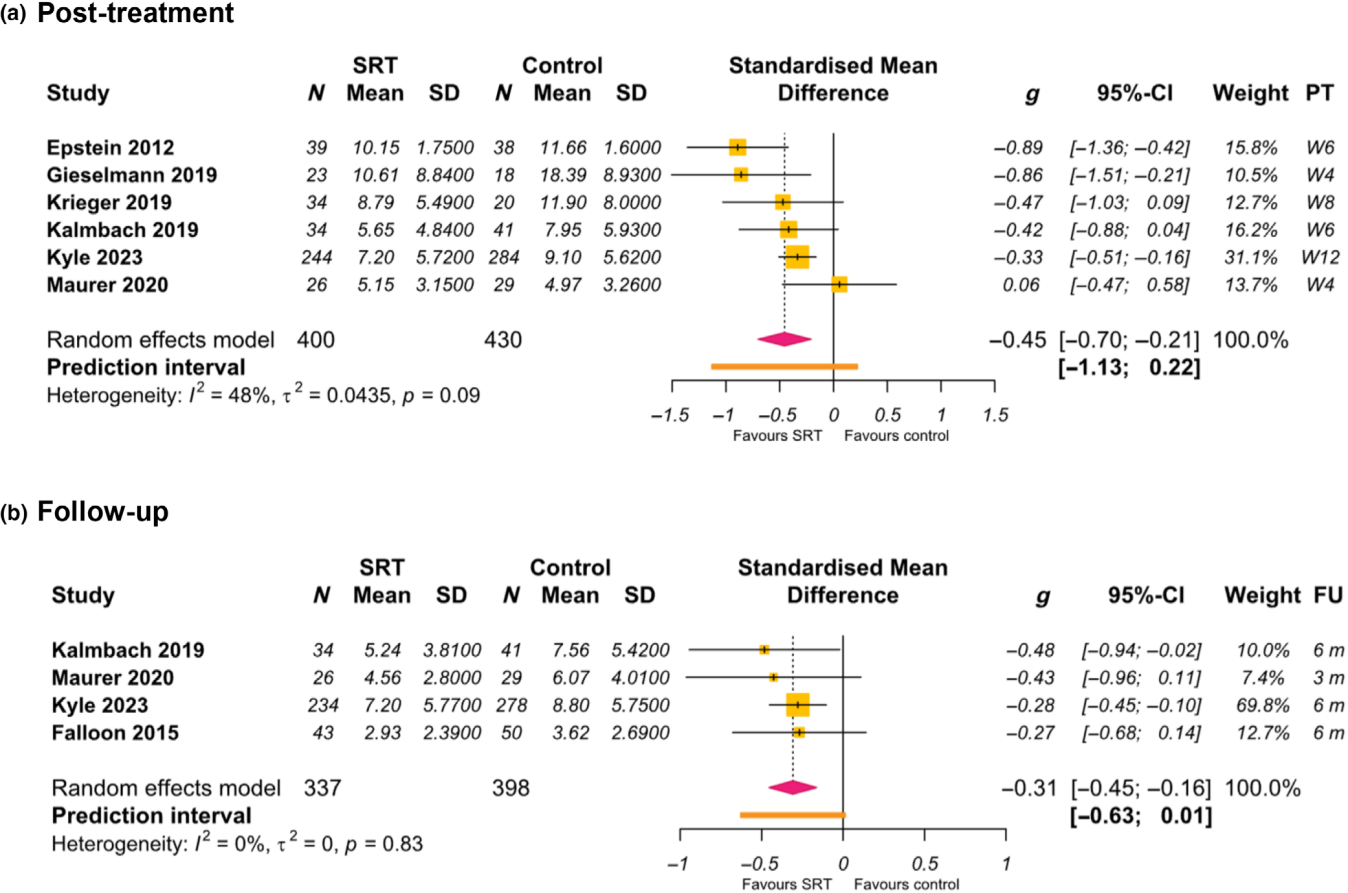
Forest plot: effects of sleep restriction therapy (SRT) on measures of depressive symptoms at post‐treatment and follow‐up.

Six studies measured depressive symptoms at post‐treatment (Epstein et al., 2012; Gieselmann & Pietrowsky, 2019; Kalmbach et al., 2019; Krieger et al., 2019; Kyle et al., 2023; Maurer et al., 2020; Figure 3a). A significant medium effect was found for reduction of depressive symptoms favouring SRT arm versus control (*N*_c_ = 6; *g* = −0.45 [95% CI = −0.70 to −0.21], *p* < 0.001). The *I* ^2^ test revealed moderate heterogeneity between studies (*I* ^2^ = 48% [95% CI = 0 to 79], *p* = 0.09).

### 4.2 At follow‐up

Four studies measured depressive symptoms at follow‐up (Falloon et al., 2015; Kalmbach et al., 2019; Kyle et al., 2023; Maurer et al., 2020; Figure 3b). A significant small effect was found for reduction of depressive symptoms favouring SRT versus control (*N*_c_ = 4; *g* = −0.31 [95% CI = −0.45 to −0.16], *p* < 0.001). The *I* ^2^ test revealed small heterogeneity between studies (*I* ^2^ = 0% [95% CI = 0 to 85], *p* = 0.83).

### 4.3 Narrative synthesis of uncontrolled studies

Aji et al. (2020) delivered a 3‐week SRT protocol via mobile application to 15 women with insomnia. Baseline score of depressive symptoms (HADS‐D) was 7.3 (SD = 4.9), which is classified as “normal”. Aji reported no significant difference between baseline and 6‐week post‐treatment, although no means and standard deviation of post‐treatment HADS score were provided in the paper.

Lancee et al. (2019) delivered 6‐week SRT to seven individuals with insomnia and depressive symptoms; six individuals completed assessments at all timepoints. Baseline scores of depressive symptoms (PHQ‐9) were 12.2 (SD = 2.3), which equates to “moderate” depression, though none of the participants were experiencing a current depressive episode at baseline (as defined by Structured Clinical Interview for DSM‐5, the Research Version). Depression severity was measured by PHQ‐9 and BDI‐II. Compared with pre‐treatment, Lancee reported in the supplementary materials that PHQ‐9 scores were significantly lower at post‐treatment (*g* = 1.66). Lancee reported no significant differences for BDI‐II at post‐treatment though a large effect was observed (*g* = 1.30). Both depression measures (PHQ‐9 and BDI‐II) were significantly lowered at 3‐month and 6‐month follow‐up compared with pre‐treatment, with large effect sizes (3 m FU: PHQ‐9, *g* = 5.40; BDI‐II, *g* = 2.80; 6 m FU: PHQ‐9, *g* = 3.17; BDI‐II, *g* = 1.95). Appendix 4 presents the summary findings of uncontrolled trials.

## 5 DISCUSSION

### 5.1 Summary

The primary objective of this review was to evaluate the impact of single‐component SRT on depressive symptoms. Meta‐analysis of RCTs revealed a medium treatment effect for SRT versus control on depressive symptoms at post‐treatment (*g* = −0.45) and a small treatment effect at follow‐up of up to 6 months (*g* = −0.31). While the number of included studies was small (post‐treatment *N*_c_ = 6; follow‐ups *N*_c_ = 4) and variability between studies (heterogeneity and prediction interval) was moderate, our findings indicate that SRT may be efficacious in improving depressive symptoms.

Our current findings are comparable in magnitude, if not slightly larger, than effects observed for full CBT‐I (Ballesio et al., 2018; Benz et al., 2020) at post‐treatment (CBT‐I individual face‐to‐face, *d* range: 0.34–0.37), although we cannot draw firm conclusions given the differences in number of trials and populations (e.g. SRT *N*_*c*_ = 7, CBT‐I *N*_*c*_ = 30; Benz et al., 2020). Related to this, Kyle et al. (2023) had the largest sample size of the meta‐analysis, representing 62% of the total number of participants at post‐treatment (total *N*_p_ = 830, Kyle *N*_p_ = 528) and 70% at follow‐up (total *N*_p_ = 735, Kyle *N*_p_ = 512). Findings of this meta‐analysis are therefore heavily weighted on this study.

Maurer et al. (2020) was the only RCT in the meta‐analysis that did not show an effect size in favour of SRT for measures of depressive symptoms at post‐treatment. One possible explanation is that their control group, TBR, matched well with SRT for therapist time, structure of treatment, and implementation and review of a weekly sleep schedule. Controlling for these non‐specific treatment effects may have attenuated group effects for depression.

### 5.2 Methodological considerations

There are several key limitations of our meta‐analysis that may affect interpretation of our findings. While we appraised study risk of bias, we did not perform a formal assessment of the certainty of evidence (through, e.g., the GRADE system). We found wide prediction intervals that stretch to the possibility of a null effect and moderate heterogeneity between studies. Having a small number of studies meant we were not able to assess publication bias or conduct subgroup analysis to explore possible causes of heterogeneity. Heterogeneity is likely due to clinical diversity, including variability in characteristics of the sample population, SRT implementation, and the different types of depression outcome measures.

Less than half of the included studies provided information on all SRT parameters. We found variability in minimum sleep window time, SE criteria and sleep window change, which may affect the mechanisms of SRT, such as homeostatic sleep pressure. Variation in SRT parameters may reflect attempts to minimise the side‐effects of SRT from excessive sleep loss, to improve treatment experience/engagement, and/or minimise drop‐out. Consistent with previous work on SRT (Kyle et al., 2015; Maurer et al., 2021), we recommend that future studies report the full SRT procedures to increase transparency.

Five different depression measures were used from the included studies, two of which did not include a sleep item (i.e. HADS, GDS). Studies where the depression measure had a sleep item may have led to larger effects, given that SRT is an effective treatment for relieving insomnia symptoms (Maurer et al., 2021). However, it should be noted that Epstein et al. (2012) used a depression measure without a sleep item (GDS) and reported one of the largest treatment effects (*g* = −0.81) in our meta‐analysis.

Five out of seven included studies were classified as high risk of bias, only two studies raised some concerns on the risk of bias. The two most affected domains in the risk of bias were trial pre‐registration and blinding outcome assessment. Only Kyle et al. (2023) pre‐registered the trial hypotheses, methods and analyses; five studies registered the trial after data collection. Registering the trial retrospectively makes it difficult to distinguish between confirmatory analyses and exploratory analyses, and creates scope for biased reporting.

The nature of behavioural sleep treatment made it difficult to blind participants and researchers from the treatment and control conditions. Non‐specific treatment effects are important to control for, particularly when self‐reported outcome measures are used (Furukawa et al., 2021). However, it is generally quite difficult to control for all non‐specific treatment effects in behavioural therapies, especially given the constraints of resource personnel, funding and time. One should therefore be aware of the possible consequences from different types of control groups when interpreting and comparing findings from behavioural therapies.

### 5.3 Broader considerations

No study examined depression as a primary outcome or specifically recruited individuals with a diagnosis of depression. Five out of seven included studies were conducted in individuals with no‐to‐mild levels of depression. Although participants in the other two studies (Epstein et al., 2012; Kyle et al., 2023) had moderate‐to‐severe level of depression at baseline, neither study had depression severity cut‐offs as their inclusion criteria; participants were simply not excluded based on depression level. The observed effects in our current findings may be underestimated, given depression severity of the sample population is not in the clinical range, creating little room for improvement. Future studies may therefore wish to recruit individuals with a diagnosis of depression to allow findings to be more translatable to practice. Consistent with previous recommendations (Edinger et al., 2021), future studies may also wish to focus on elucidating possible side‐effects of time in bed restriction during SRT in a sample with clinical depression.

There is a need for future mechanistic work to examine how insomnia reduction through SRT drives improvement in depression. Individuals with insomnia report high levels of maladaptive cognitive patterns linked to depression, including worry (Harvey, 2000; Jansson & Linton, 2006; Jansson‐Fröjmark et al., 2012; Mitchell et al., 2012), rumination (Carney et al., 2006; Mitchell et al., 2012; Thomsen et al., 2003) and emotional dysregulation (Galbiati et al., 2020; Palagini et al., 2017). Being awake at night, where there is typically a lack of visual and auditory stimuli, may be conducive to intrusive and ruminative thoughts (Lovato & Gradisar, 2014) and fuel negatively toned cognitive activity, thus further disrupting sleep. These bidirectional interactions are likely self‐sustaining and may develop into or exacerbate depressive symptoms overtime (McLaughlin et al., 2007). During SRT, the sleep window is restricted, which directly strengthens homeostatic sleep drive (Maurer et al., 2018) and reduces wake‐time in bed (Maurer et al., 2021; Simon et al., 2023). Improvement in sleep continuity and sleep depth may also have direct effects on daytime mood, worry (Harvey, 2000; Jansson & Linton, 2006; Jansson‐Fröjmark et al., 2012; Mitchell et al., 2012), rumination (Carney et al., 2006; Mitchell et al., 2012; Thomsen et al., 2003) and emotional processing (Galbiati et al., 2020; Palagini et al., 2017). Regularising bedtime and rise times likely engenders more regular light exposure, social rhythm and potentially behavioural activation, which may feedback to reinforce good sleep quality, daytime functioning and reduce depressive symptoms. These pathways require dedicated evaluation in future studies.

## AUTHOR CONTRIBUTIONS

**Katrina Yan Kei Tse:** Conceptualization; investigation; writing – original draft; writing – review and editing; visualization; methodology; formal analysis; software; data curation; project administration; validation. **Leonie Franziska Maurer:** Supervision; conceptualization; writing – review and editing; investigation. **Colin Alexander Espie:** Supervision; writing – review and editing; conceptualization; funding acquisition. **Simon David Kyle:** Supervision; conceptualization; writing – review and editing; funding acquisition.

## FUNDING INFORMATION

KYT, CAE and SDK are supported by the NIHR Oxford Health Biomedical Research Centre (NIHR203316).

## CONFLICT OF INTEREST STATEMENT

The authors declare no conflicts of interest.

## Supporting information


**DATA S1.** Supporting Information.

## Data Availability

Data sharing is not applicable to this article as no new data were created or analyzed in this study.
