# Supplementary material for: The effect of single‐component sleep restriction therapy on depressive symptoms: A systematic review and meta‐analysis
Source: J Sleep Res. 2024 Feb 28;33(6):e14180. doi: 10.1111/jsr.14180 (PMC11596993; doi:10.1111/jsr.14180)
Supplement: Supplementary file 1 — DATA S1. Supporting Information. [file JSR-33-e14180-s001.docx]

## **Appendix 1**

### ***Breakdown of SRT procedures***

**Supplementary Table 1.** The breakdown of SRT procedures from included studies

| Study ID | Sleep window generation | Minimum TIB | SE criteria & Change to sleep window | Position of sleep window |
| --- | --- | --- | --- | --- |
| Kyle 2023 ^1^ | Self-reported TST over last week (sleep diary) | 5 hrs | a) SE ≥ 85% => TIB + 15 min,  b) SE < 80% => TIB – 15 min,  c) SE 80%–84% => No change to TIB | Rise time typically aligns with working schedule; Bedtime is typically delayed |
| Maurer 2020  ^2^ | Self-reported TST over last 2 weeks (sleep diary) | 5 hrs | a) SE ≥ 90% => TIB + 15 min,  b) SE < 85% => TIB – 15 min,  c) SE 85%–89% => No change to TIB | Patient preference |
| Krieger 2019  ^3^ | Not reported | 6 hrs | SE ≥ 90% => TIB + 30 min | Patient preference |
| Kalmbach 2019  ^4^ | Not reported | Not reported | Not reported | Not reported |
| Gieselmann 2019  ^5^ | Self-reported TST over last week (sleep diary) | Not reported | SE > 85% => TIB + 15 min | Not reported |
| Falloon 2015  ^6^ | Self-reported TST + 50% of time spent awake in bed over last 2 weeks (sleep diary) | 5 hrs | SE < 85% => Baseline TST + 30 mins | Patient preference |
| Epstein 2012  ^7^ | Self-reported TST over last 2 weeks (sleep diary) | 5 hrs | a) SE ≥ 90% => TIB + 15 min,  b) SE < 85% => TIB – 15 min,  c) SE 85%–89% => No change to TIB | Patient preference |
| Aji 2020  ^8^ | Self-reported TST (questionnaire) | 5.5 hr | Not reported, 15 mins | Not reported |
| Lancee 2019  ^9^ | Self-reported TST in the past week | 5 hrs | SE > 85%, Not reported, | Not reported |
| Abbreviations: TST, total sleep time; TIB, time in bed; SE, sleep efficiency. | | | | |

## **Appendix 2**

### ***Baseline score for measure of depressive symptoms***

**Supplementary Table 2.** Baseline score for measure of depressive symptoms

| Study ID | Measure of depressive symptoms (contains sleep items?) | Baseline | |
| --- | --- | --- | --- |
|  |  | SRT | Control |
|  |  | Mean (SD) [n] “Scoring” | Mean (SD) [n] “Scoring” |
| Kyle 2023 ^1^ | PHQ -9 (Y) | 10.4 (5.3) [321] “Moderate Depression” | 10.1 (5.3) [321] “Moderate Depression” |
| Maurer 2020 ^2^ | HADS-D (N) | 6.15 (3.04) [27] “Normal” | 6.72 (3.54) [29] “Normal” |
| Krieger 2019 ^3^ | ADS-K (Y) | 13.05 (5.42) [41] “Normal” | 13.67 (6.69) [21] “Normal” |
| Kalmbach 2019 ^4^ | BDI-II (Y) | 6.77 (4.15) [34] “Normal” | 8.8 (5.62) [41] “Normal” |
| Gieselmann 2019 ^5^ | CES-D (Y) | 13.04 (6.85) [27] “Normal” | 18.05 (7.42) [22] “Mild Depression” |
| Falloon 2015 ^6^ | PHQ-9 (Y) | 4.98 (2.76) [46] “Normal” | 5.31 (2.8) [51] “Mild Depression” |
| Epstein 2012 ^7^ | GDS (N) | 11.11 (2.41) [44] “Moderate Depression” | 12.24 (34.18*) [50] “Severe Depression” |

Abbreviations: HADS-D, Hospital Anxiety and Depression Scale - Depression Subscale; ADS-K, Allgemeine Depressions-Skala - Kurzform (German version of CES-D); CES-D, Center for Epidemiological Studies Depression Scale; BDI-II, Beck Depression Inventory II; PHQ9-D, Patient Health Questionnaire - Depression Scale; GDS, Geriatric Depression Scale; Y, yes; N, no.

Scale developers’ scoring categories: HADS-D, 0-7: Normal; 8-10: Borderline abnormal; 11-21: Abnormal. ADS-K, >16: Risk for clinical depression. BDI-II, 0-13: Minimal; 14-19: Mild; 20-28: Moderate; 29-63: Severe. CES-D, >16: Risk for clinical depression. PHQ-9-D, 0-4: None; 5-9: mild; 10-14: Moderate; 15-19: Moderately severe; 20-27: Severe. GDS, 0-4: Normal; 5-8: Mild depression; 9-11: Moderate depression; 12-15: Severe depression.

*Note, we consider this SD to be implausible because it is larger than the range of possible scores. We contacted the corresponding author, but they did not have access to the data to confirm if the value was a typographical error.

## **Appendix 3**

### ***Revised Cochrane risk-of-bias tool for randomized trials (RoB2)– Item Breakdown***

|  | | Kyle 2023 | Maurer 2020 | Krieger 2019 | Kalmbach 2019 | Gieselmann 2019 | Falloon 2015 | Epstein 2012 |
| --- | --- | --- | --- | --- | --- | --- | --- | --- |
| 1. Randomization Process | | L | L | L | L | H | L | L |
|  | 1.1 Was the allocation sequence random? | Y | Y | Y | Y | Y | Y | Y |
|  | 1.2 Was the allocation sequence concealed until participants were enrolled and assigned to interventions? | Y | Y | Y | Y | N | Y | Y |
|  | 1.3 Did baseline differences between intervention groups suggest a problem with the randomization process? | N | N | N | N | Y | N | N |
| 2. Deviations from the Intended Interventions | | L | L | L | S | L | L | L |
|  | 2.1. Were participants aware of their assigned intervention during the trial? | Y | Y | Y | PY | Y | Y | Y |
|  | 2.2. Were carers and people delivering the interventions aware of participants' assigned intervention during the trial? | Y | Y | Y | Y | Y | Y | Y |
|  | 2.3. If Y/PY/NI to 2.1 or 2.2: Were there deviations from the intended intervention that arose because of the trial context? | PN | N | PN | PN | PN | N | N |
|  | 2.4 If Y/PY to 2.3: Were these deviations likely to have affected the outcome? | NA | NA | NA | NA | NA | NA | NA |
|  | 2.5. If Y/PY/NI to 2.4: Were these deviations from intended intervention balanced between groups? | NA | NA | NA | NA | NA | NA | NA |
|  | 2.6 Was an appropriate analysis used to estimate the effect of assignment to intervention? | Y | Y | Y | PN | Y | PY | Y |
|  | 2.7 If N/PN/NI to 2.6: Was there potential for a substantial impact (on the result) of the failure to analyse participants in the group to which they were randomized? | NA | NA | NA | N | NA | NA | NA |
| 3. Missing Outcome Data | | L | L | L | H | L | L | S |
|  | 3.1 Were data for this outcome available for all, or nearly all, participants randomized? | N | Y | N | N | PY | Y | N |
|  | 3.2 If N/PN/NI to 3.1: Is there evidence that the result was not biased by missing outcome data? | Y | NA | Y | PN | NA | NA | N |
|  | 3.3 If N/PN to 3.2: Could missingness in the outcome depend on its true value? | NA | NA | NA | NI | NA | NA | PY |
|  | 3.4 If Y/PY/NI to 3.3: Is it likely that missingness in the outcome depended on its true value? | NA | NA | NA | NI | NA | NA | PN |
| 4. Measurement of the Outcome | | S | L | H | S | H | H | H |
|  | 4.1 Was the method of measuring the outcome inappropriate? | N | N | N | N | N | N | N |
|  | 4.2 Could measurement or ascertainment of the outcome have differed between intervention groups? | N | N | N | N | N | N | N |
|  | 4.3 If N/PN/NI to 4.1 and 4.2: Were outcome assessors aware of the intervention received by study participants? | Y | Y | Y | Y | Y | Y | Y |
|  | 4.4 If Y/PY/NI to 4.3: Could assessment of the outcome have been influenced by knowledge of intervention received? | Y | PN | Y | Y | Y | Y | Y |
|  | 4.5 If Y/PY/NI to 4.4: Is it likely that assessment of the outcome was influenced by knowledge of intervention received? | PN | NA | Y | PN | Y | Y | Y |
| 5. Selection of the Reported Result | | L | S | S | S | S | S | S |
|  | 5.1 Were the data that produced this result analysed in accordance with a pre-specified analysis plan that was finalized before unblinded outcome data were available for analysis? | Y | NI | NI | PN | NI | NI | NI |
|  | Is the numerical result being assessed likely to have been selected, on the basis of the results, from… 5.2. ... multiple eligible outcome measurements (e.g. scales, definitions, time points) within the outcome domain? | N | N | N | PN | N | N | N |
|  | 5.3 ... multiple eligible analyses of the data? | N | N | N | N | N | N | N |
| Overall Judgement | | S | S | H | H | H | H | H |
| Abbreviations: Y = Yes; PY = Probably Yes; N = No; PN = Probably No; NI = No Information; NA = Not Applicable; L = Low risk of bias; S = Some risk of bias; H = High risk of bias. Colour codes: Green = Low risk of bias; Yellow = Some risk of bias; Red = High risk of bias. Note that the colour change represents the point of which the answer changed the risk of bias. | | | | | | | | |


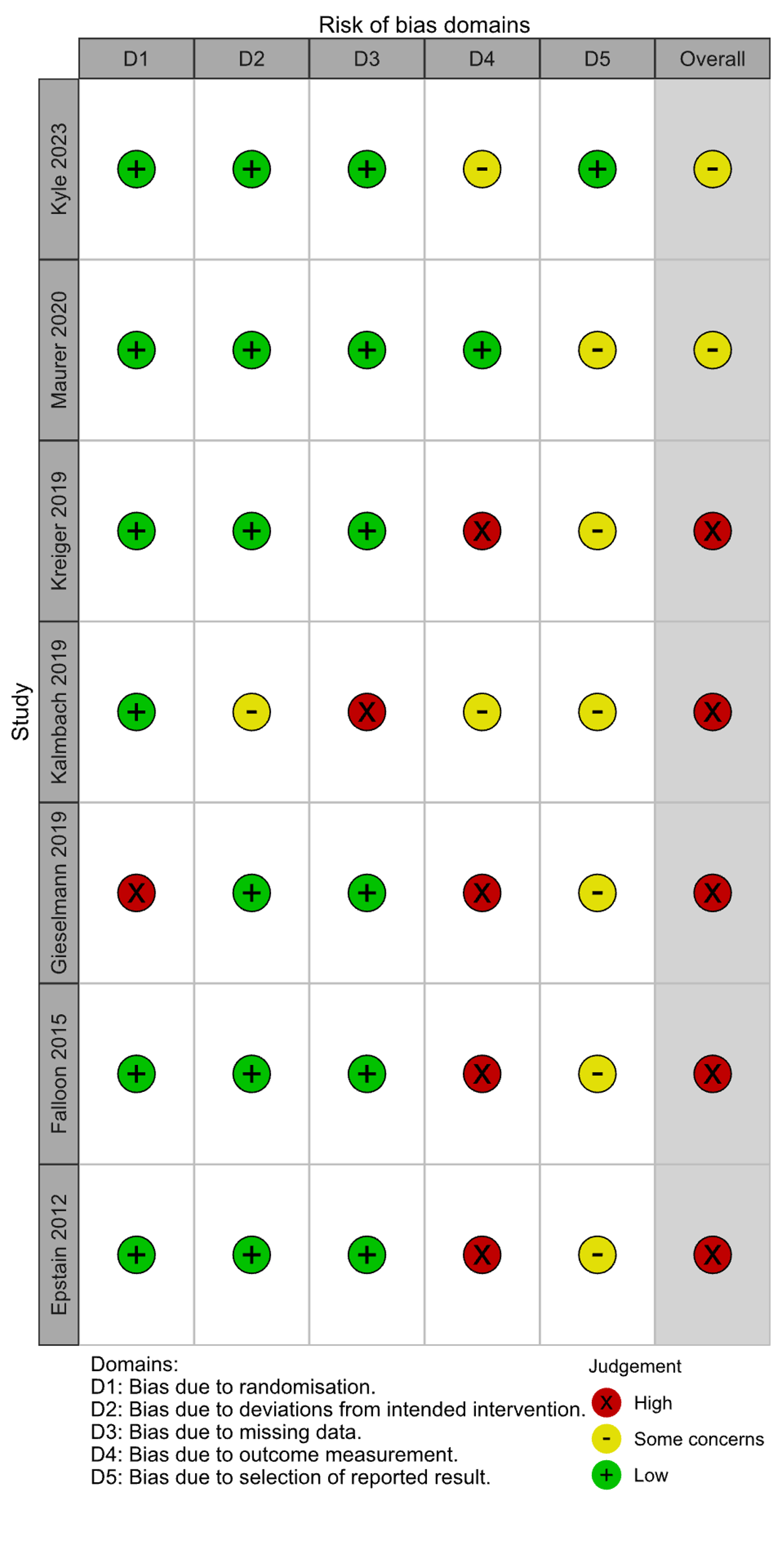


**Supplementary Figure 1.** Risk of bias summary

## **Appendix 4**

### ***Summary findings of uncontrolled trials***

**Supplementary Table 3.** Summary findings for measure of depressive symptoms from UCTs.

| Study ID | Sample size (N) | Findings at post-treatment compared to baseline | Findings at follow-up compared to baseline |
| --- | --- | --- | --- |
| Aji 2020  ^8^ | 15 | W6: no change in HADS scores (*Z* = −1.8, *p* = .07)*. | / |
| Lancee 2019  ^9^ | 7 | W8: decrease in PHQ-9 scores (baseline mean (*SD*) = 12.5 (1.05), post-treatment mean (*SD*) = 7.33 (4.18), *g* = 1.66, *p* < .01), but no change in BDI-II scores (baseline mean (*SD*) = 19.2 (3.19), post-treatment mean (*SD*) = 13.3 (4.93), *g* = 1.30, *p* =.11) | Depressive symptoms, as measured by PHQ-9, continued to decrease at 3-month (PHQ-9: mean (*SD*) = 4.17 (1.72), *g* = 5.40, *p* < .001; BDI-II: mean (*SD*) = 7.67 (4.32), *g* = 2.80, *p* = .006) and 6-month (PHQ-9: mean (*SD*) = 6.67 (2.16), *g* = 3.17, *p* < .001; BDI-II: mean (*SD*) = 10.2 (5.12), *g* = 1.95, *p* <.05) |

Abbreviations: HADS, Hospital Anxiety and Depression Scale; PHQ-9, Patient Health Questionnaire.

* No means (*SD*) reported at post-treatment.

## **Appendix 5**

### ***The effect of sleep restriction therapy on mood-related outcomes***

Supplementary Table 4 summarises the characteristics of the study and Supplementary Table 5 presents the breakdown of SRT procedures. Miller et al. (2013)^10^ found significantly lower positive mood at week 1 of SRT (indicating initial deteriorations of positive mood), and a trend for improvement in positive mood at week 3 of SRT. There was no significant difference in negative mood at week 1 of SRT compared to baseline, but there was a significant reduction in negative mood at week 3 of SRT compared to baseline (indicating symptoms improvement through SRT).

**Supplementary Table 4.** Characteristics of the study

| Author, Year, *Location* | Study Design | Sample size randomised (female), Mean age (SD), Recruitment | Hypnotic medication status, Co-morbidity | Diagnostic Criteria of Insomnia (*determined by*) | Intervention & its format | Depression measure (PT, FU) |
| --- | --- | --- | --- | --- | --- | --- |
| Miller 2013  *UK* ^10^ | UCT | N = 9 (6 f) 46.4 yrs (range: 34-58) Adults from community | Medication free No comorbidity reported | Research Diagnostic Criteria for Psychophysiological insomnia (*2 nights of PSG*) | 3-wk SRT Individual, FtF (3 sessions), Phone (two calls) | DISS – negative mood (W4, /) |
| Abbreviations: UCT, Uncontrolled clinical trial; SRT, Sleep Restriction Therapy; DISS, Daytime Insomnia Symptoms Scale; FtF, face to face; PSG, polysomnography; PT, post treatment; FU, follow up. | | | | | | |

**Supplementary Table 5.** The breakdown of SRT procedures

| Study ID | Sleep window generation | Minimum TIB | SE criteria & Change to sleep window | Position of sleep window |
| --- | --- | --- | --- | --- |
| Miller 2013 ^10^ | Not reported | 5 hrs | a) SE ≥ 90% => TIB + 15 min,  b) SE < 85% => TIB – 15 min,  c) SE 85%–89% => No change to TIB | Not reported |
| Abbreviations: TST, total sleep time; TIB, time in bed; SE, sleep efficiency. | | | | |

## **Appendix 6**

### ***The full line by line search strategy in each database***

Web of Knowledge

| #9 | (#4) AND (#8) |
| --- | --- |
| #8 | #5 OR #6 OR #7 |
| #7 | (AK = ("sleep restrict*" OR "sleep compress*" OR "bedtime restriction" OR "time in bed restriction" ) ) *AND***LANGUAGE:** (English) *AND* **DOCUMENT TYPES:** (Article)  *Indexes=SCI-EXPANDED, SSCI, A&HCI, CPCI-S, CPCI-SSH, BKCI-S, BKCI-SSH, ESCI, CCR-EXPANDED, IC Timespan=All years* |
| #6 | (AB = ("sleep restrict*" OR "sleep compress*" OR "bedtime restriction" OR "time in bed restriction" ) ) *AND***LANGUAGE:** (English) *AND* **DOCUMENT TYPES:** (Article)  *Indexes=SCI-EXPANDED, SSCI, A&HCI, CPCI-S, CPCI-SSH, BKCI-S, BKCI-SSH, ESCI, CCR-EXPANDED, IC Timespan=All years* |
| #5 | (TI = ("sleep restrict*" OR "sleep compress*" OR "bedtime restriction" OR "time in bed restriction" ) ) *AND***LANGUAGE:** (English) *AND* **DOCUMENT TYPES:** (Article)  *Indexes=SCI-EXPANDED, SSCI, A&HCI, CPCI-S, CPCI-SSH, BKCI-S, BKCI-SSH, ESCI, CCR-EXPANDED, IC Timespan=All years* |
| #4 | #1 OR #2 OR #3 |
| #3 | (AK = ("insomnia*" OR "sleep disorder" OR "sleep disturbance " ) ) *AND***LANGUAGE:** (English) *AND* **DOCUMENT TYPES:** (Article)  *Indexes=SCI-EXPANDED, SSCI, A&HCI, CPCI-S, CPCI-SSH, BKCI-S, BKCI-SSH, ESCI, CCR-EXPANDED, IC Timespan=All years* |
| #2 | (AB = ("insomnia*" OR "sleep disorder" OR "sleep disturbance " ) ) *AND***LANGUAGE:** (English) *AND* **DOCUMENT TYPES:** (Article)  *Indexes=SCI-EXPANDED, SSCI, A&HCI, CPCI-S, CPCI-SSH, BKCI-S, BKCI-SSH, ESCI, CCR-EXPANDED, IC Timespan=All years* |
| #1 | (TI = ("insomnia*" OR "sleep disorder" OR "sleep disturbance " ) ) *AND***LANGUAGE:** (English) *AND* **DOCUMENT TYPES:** (Article)  *Indexes=SCI-EXPANDED, SSCI, A&HCI, CPCI-S, CPCI-SSH, BKCI-S, BKCI-SSH, ESCI, CCR-EXPANDED, IC Timespan=All years* |

PubMed

| #3 | (#1) AND (#2) |
| --- | --- |
| #2 | ((insomnia*[Title/Abstract]) OR (sleep disorder[Title/Abstract])) OR (sleep disturbance[Title/Abstract]) Filters: Clinical Study, Clinical Trial, Controlled Clinical Trial, Randomized Controlled Trial, Humans, English, Adult: 19+ years |
| #1 | (((sleep restrict*[Title/Abstract]) OR (sleep compress*[Title/Abstract])) OR (bedtime restriction[Title/Abstract])) OR (time in bed restriction[Title/Abstract]) Filters: Clinical Study, Clinical Trial, Controlled Clinical Trial, Randomized Controlled Trial, Humans, English, Adult: 19+ years |

SCOPUS

| #1 | TITLE-ABS-KEY ( ( ( "insomnia*" )  OR  ( "sleep disorder" )  OR  ( "sleep disturbance" ) )  AND  ( ( "sleep restrict*" )  OR  ( "sleep compress*" )  OR  ( "bedtime restriction" )  OR  ( "time in bed restriction" ) ) )  AND  (  LIMIT-TO ( ACCESSTYPE(OA) ) )  AND  ( LIMIT-TO ( DOCTYPE ,  "ar" ) )  AND  ( LIMIT-TO ( LANGUAGE ,  "English" ) )  AND  ( LIMIT-TO ( SRCTYPE ,  "j" ) ) |
| --- | --- |

CENTRAL

| #1 | ( ( ( "insomnia*" ) OR ( "sleep disorder" ) OR ( "sleep disturbance" ) ) AND ( ( "sleep restrict*" ) OR ( "sleep compress*" ) OR ( "bedtime restriction" ) OR ( "time in bed restriction" ) ) ) in Title Abstract Keyword - with Publication Year from 1986 to 2020, with Cochrane Library publication date Between Jan 1986 and Aug 2023, in Trials (Word variations have been searched) |
| --- | --- |

PsychINFO

| #1 | ab(((("insomnia*") OR ("sleep disorder") OR ("sleep disturbance")) AND (("sleep restrict*") OR ("sleep compress*") OR ("bedtime restriction") OR ("time in bed restriction")))) OR ti(((("insomnia*") OR ("sleep disorder") OR ("sleep disturbance")) AND (("sleep restrict*") OR ("sleep compress*") OR ("bedtime restriction") OR ("time in bed restriction")))) |
| --- | --- |

## **Appendix 7**

### ***Excluded studies from full text screening***

The hierarchy of the exclusion reasons mirrors the order of the inclusion criteria, as follows:

1. Study was a conference abstract.
2. Study was not conduct in the adult population.
3. Sample did not fulfil the criteria for insomnia disorder.
4. Study did not implement a standalone SRT intervention.
5. Study did not include measure of depressive symptoms.
6. Study was not RCT or UCT.
7. RCT did not include permitted control conditions, as stated in inclusion criteria.

**Supplementary Table 6.** List of excluded studies from full-text screening

| Reason for exclusion | Conference abstract | Adults | Insomnia | SRT | Depression measure | RCT or UCT | Control |
| --- | --- | --- | --- | --- | --- | --- | --- |
| Morin 1990 ^11^ | N | Y | Y | Y | N |  |  |
| Friedman 1991 ^12^ | N | Y | Y | Y | N |  |  |
| Riedel 1995 ^13^ | N | Y | Y | Y | N |  |  |
| Bliwise 1995 ^14^ | N | Y | Y | Y | N |  |  |
| Lichstein 2001 ^15^ | N | Y | Y | N |  |  |  |
| Riedel 2001 ^16^ | N | Y | Y | Y | N |  |  |
| Vincent 2008 ^17^ | N | Y | Y | N |  |  |  |
| Falloon 2010 ^18^ | Y |  |  |  |  |  |  |
| Taylor 2010 ^19^ | N | Y | Y | Y | N |  |  |
| Marin 2011 ^20^ | Y |  |  |  |  |  |  |
| Kyle 2011 ^21^ | N | Y | Y | Y | N |  |  |
| An 2012 ^22^ | N | Y | Y | N |  |  |  |
| Vallières 2013 ^23^ | N | Y | Y | Y | N |  |  |
| Falloon 2013 ^24^ | N | Y | Y | Y | N |  |  |
| Kyle 2013 ^25^ | N | Y | Y | Y | N |  |  |
| Miller 2013 ^10^ | N | Y | Y | Y | N |  |  |
| Antonio 2013 ^26^ | N | Y | Y | Y | N |  |  |
| Kyle 2014 ^27^ | N | Y | Y | Y | N |  |  |
| Morin 2015 ^28^ | Y |  |  |  |  |  |  |
| Anderson 2015 ^29^ | Y |  |  |  |  |  |  |
| Graham 2015 ^30^ | Y |  |  |  |  |  |  |
| Wang 2015 ^31^ | N | Y | Y | N |  |  |  |
| Duss 2016 ^32^ | N | Y | Y | N |  |  |  |
| Hyde-Nolan 2017 ^33^ | Y |  |  |  |  |  |  |
| Judge 2017 ^34^ | Y |  |  |  |  |  |  |
| Cheng 2017 ^35^ | Y |  |  |  |  |  |  |
| Sidani 2018 ^36^ | N | Y | Y | Y | N |  |  |
| Tonnu 2018 ^37^ | Y |  |  |  |  |  |  |
| Arnedt 2018 ^38^ | Y |  |  |  |  |  |  |
| Rosen 2018 ^39^ | Y |  |  |  |  |  |  |
| D'Onofrio 2018 ^40^ | Y |  |  |  |  |  |  |
| Tallent 2018 ^41^ | Y |  |  |  |  |  |  |
| Whittall 2018 ^42^ | N | Y | Y | Y | N |  |  |
| Drake 2019 ^43^ | N | Y | Y | Y | N |  |  |
| Maurer 2019 ^44^ | Y |  |  |  |  |  |  |
| Maurer 2019 ^45^ | Y |  |  |  |  |  |  |
| Sidani 2019 ^46^ | N | Y | Y | Y | N |  |  |
| Abbreviations: N = No; Y = Yes. | | | | | | | |

## **References**

1. Kyle, S. D. *et al.* Clinical and cost-effectiveness of nurse-delivered sleep restriction therapy for insomnia in primary care (HABIT): a pragmatic, superiority, open-label, randomised controlled trial. *The Lancet* (2023) doi:10.1016/S0140-6736(23)00683-9.

2. Maurer, L. F. *et al.* Isolating the role of time in bed restriction in the treatment of insomnia: A randomized, controlled, dismantling trial comparing sleep restriction therapy with time in bed regularization. *Sleep* **43**, (2020).

3. Krieger, T. *et al.* A randomized controlled trial comparing guided internet-based multi-component treatment and internet-based guided sleep restriction treatment to care as usual in insomnia. *Sleep Med* **62**, 43–52 (2019).

4. Kalmbach, D. A. *et al.* Treating insomnia improves depression, maladaptive thinking, and hyperarousal in postmenopausal women: comparing cognitive-behavioral therapy for insomnia (CBTI), sleep restriction therapy, and sleep hygiene education. *Sleep Med* **55**, 124–134 (2019).

5. Gieselmann, A. & Pietrowsky, R. The effects of brief chat-based and face-to-face psychotherapy for insomnia: a randomized waiting list controlled trial. *Sleep Med* **61**, 63–72 (2019).

6. Falloon, K., Raina Elley, C., Fernando, A., Lee, A. C. & Arroll, B. Simplified sleep restriction for insomnia in general practice: A randomised controlled trial. *British Journal of General Practice* **65**, e508–e515 (2015).

7. Epstein, D. R., Sidani, S., Bootzin, R. R. & Belyea, M. J. Dismantling multicomponent behavioral treatment for insomnia in older adults: A randomized controlled trial. *Sleep* **35**, 797–805 (2012).

8. Aji, M. *et al.* A feasibility study of a mobile app to treat insomnia. *Transl Behav Med* **11**, 604–612 (2020).

9. Lancee, J., Maric, M. & Kamphuis, J. H. Sleep restriction therapy may be effective for people with insomnia and depressive complaints : evidence from a case series. *Behavioural and Cognitive Psychotherapy* **48**, 1–6 (2019).

10. Miller, C. B., Kyle, S. D., Marshall, N. S. & Espie, C. A. Ecological momentary assessment of daytime symptoms during sleep restriction therapy for insomnia. *J Sleep Res* **22**, 266–272 (2013).

11. Morin, C. M., Kowatch, R. A. & O’Shanick, G. Sleep restriction for the inpatient treatment of insomnia. *Sleep* **13**, 183–186 (1990).

12. Friedman, L., Bliwise, D. L., Yesavage, J. A. & Salom, S. R. A preliminary study comparing sleep restriction and relaxation treatments for insomnia in older adults. *Journals of Gerontology* **46**, 8 (1991).

13. Riedel, B. W., Lichstein, K. L. & Dwyer, W. O. Sleep compression and sleep education for older insomniacs: Self-help versus therapist guidance. *Psychol Aging* **10**, 54–63 (1995).

14. Bliwise, D. L., Friedman, L., Nekich, J. C. & Yesavage, J. A. Prediction of outcome in behaviorally based insomnia treatments. *J Behav Ther Exp Psychiatry* **26**, 17–23 (1995).

15. Lichstein, K. L., Riedel, B. W., Wilson, N. M., Lester, K. W. & Aguillard, R. N. Relaxation and sleep compression for late-life insomnia: A placebo-controlled trial. *J Consult Clin Psychol* **69**, 227–239 (2001).

16. Riedel, B. W. & Lichstein, K. L. Strategies for evaluating adherence to sleep restriction treatment for insomnia. *Behaviour Research and Therapy* **39**, 201–212 (2001).

17. Vincent, N., Lewycky, S. & Finnegan, H. Barriers to Engagement in Sleep Restriction and Stimulus Control in Chronic Insomnia. *J Consult Clin Psychol* **76**, 820–828 (2008).

18. Falloon, K. Refresh: restriction for reorganisation of sleep habit. Protocol for a randomised controlled trial of bedtime restriction for the treatment of primary insomnia in the primary care setting. *Sleep Biol Rhythms* **8**, A41‐ (2010).

19. Taylor, D. J., Schmidt-Nowara, W., Jessop, C. A. & Ahearn, J. *Sleep restriction therapy and hypnotic withdrawal versus sleep hygiene education in hypnotic using patients with insomnia*. *Journal of Clinical Sleep Medicine* vol. 6 (2010).

20. Marin, H. A. Cognitive-behavioral treatment of chronic insomnia. Randomized study in a sample of colombian patients. *Sleep* **34**, A189 (2011).

21. Kyle, S. D., Morgan, K., Spiegelhalder, K. & Espie, C. A. No pain, no gain: An exploratory within-subjects mixed-methods evaluation of the patient experience of sleep restriction therapy (SRT) for insomnia. *Sleep Med* **12**, 735–747 (2011).

22. An, H., Park, J., Jang, E. S. & Chung, S. The impact of temperament and character on the efficacy of nonpharmacologic treatment of primary insomnia. *Compr Psychiatry* **53**, 201–207 (2012).

23. Vallières, A., Ceklic, T., Bastien, C. H. & Espie, C. A. A Preliminary Evaluation of the Physiological Mechanisms of Action for Sleep Restriction Therapy. *Sleep Disord* **2013**, 1–15 (2013).

24. Falloon, K., Elley, C. R., Fernando III, A., Lee, A. & Arroll, B. Refresh: restriction for reorganising sleep habit-a randomised controlled trial of sleep restriction for primary insomnia. *Sleep Biol Rhythms* **11**, 1‐2 (2013).

25. Kyle, S. D. *et al.* The Glasgow Sleep Impact Index (GSII): a novel patient-centred measure for assessing sleep-related quality of life impairment in Insomnia Disorder. *Sleep Med* **14**, 493–501 (2013).

26. Antonio, F., Arroll, B. & Falloon, K. A double-blind randomised controlled study of a brief intervention of bedtime restriction for adult patients with primary insomnia. *J Prim Health Care* **5**, 5–10 (2013).

27. Kyle, S. *et al.* Sleep Restriction Therapy for Insomnia is Associated with Reduced Objective Total Sleep Time, Increased Daytime Somnolence, and Objectively Impaired Vigilance: Implications for the Clinical Management of Insomnia Disorder. *Sleep* **37**, 229–237 (2014).

28. Morin, C. M. *et al.* Long-term efficacy of cognitive behavior therapy, behavior therapy, and cognitive therapy for chronic insomnia. *Sleep* **38**, A224 (2015).

29. Anderson, J. R. *et al.* Efficacy of cognitive behavioral therapy for insomnia and sleep restriction therapy for postmenopausal insomnia and comorbid vasomotor symptoms. *Sleep* **38**, A229--A230 (2015).

30. Graham, L., Lovato, N. & Lack, L. The use of brief daytime naps in the behavioural treatment of chronic primary insomnia. *Sleep Biol Rhythms* **13**, 63 (2015).

31. Wang, J., Yin, G., Li, G., Liang, W. & Wei, Q. Efficacy of physical activity counseling plus sleep restriction therapy on the patients with chronic insomnia. *Neuropsychiatr Dis Treat* **11**, 2771–2778 (2015).

32. Duss, S. B. *et al.* i-Sleep: internet-based intervention against insomnia. *J Sleep Res* **25**, 120‐ (2016).

33. Hyde-Nolan, M. *et al.* 0359 Efficacy of Behavioral Insomnia Treatment on Post-Menopausal Quality of Life. *Sleep* **40**, A133–A134 (2017).

34. Judge, D. *et al.* Sleep restriction therapy + armodafinil for insomnia disorder (a pilot study for the moderate trial); trial protocol and progress update. *J Sleep Res* **26**, 38‐ (2017).

35. Cheng, P. *et al.* 1086 Cbt-I for Menopause Related Insomnia Also Reduces Depression Severity. *Sleep* **40**, A405–A405 (2017).

36. Sidani, S., Epstein, D. R., Fox, M. & Collins, L. The contribution of participant, treatment, and outcome factors to treatment satisfaction. *Res Nurs Health* **41**, 572–582 (2018).

37. Tonnu, C. V *et al.* 0706 Impact of Behavioral Insomnia Treatment on Post-Menopausal Female Sexual Functioning. *Sleep* **41**, A262–A263 (2018).

38. Arnedt, J. T. *et al.* 0394 Efficacy of Cognitive Behavioral Therapy for Insomnia on Post-Menopausal Quality of Life. *Sleep* **41**, A150–A150 (2018).

39. Rosen, A. *et al.* Cognitive behavior therapy for insomnia-is sleep compression an equally effective and less difficult alternative compared to sleep restriction? *J Sleep Res* **27**, 409‐ (2018).

40. D’Onofrio, P., Jernelov, S., Rosen, A., Kaldo, V. & Akerstedt, T. Polysomnographical effects of on-line cognitive behavioral therapy for insomnia. *J Sleep Res* **27**, 408‐409 (2018).

41. Tallent, G. *et al.* 0397 Impact of Behavioral Insomnia Treatments on Quality of Life in Post-Menopausal Women. *Sleep* **41**, A151–A151 (2018).

42. Whittall, H., Pillion, M. & Gradisar, M. Daytime sleepiness, driving performance, reaction time and inhibitory control during sleep restriction therapy for Chronic Insomnia Disorder. *Sleep Med* **45**, 44–48 (2018).

43. Drake, C. L. *et al.* Treating chronic insomnia in postmenopausal women: A randomized clinical trial comparing cognitive-behavioral therapy for insomnia, sleep restriction therapy, and sleep hygiene education. *Sleep* **42**, 1–11 (2019).

44. Maurer, L. F., Espie, C. A., Omlin, X. & Kyle, S. D. 0385 Daytime Impairment during Sleep Restriction Therapy for Insomnia: Results from a Randomised-Controlled Trial. *Sleep* **42**, A156–A156 (2019).

45. Maurer, L. F., Espie, C. A., Omlin, X. & Kyle, S. D. Is restriction of time in bed central to the efficacy of sleep restriction therapy for insomnia? results from a randomised, controlled, dismantling trial comparing sleep restriction with bedtime consistency. *Sleep Med* **64**, S248 (2019).

46. Sidani, S., Epstein, D. R., Fox, M. & Collins, L. Comparing the Effects of Single- and Multiple-Component Therapies for Insomnia on Sleep Outcomes. *Worldviews Evid Based Nurs* **16**, 195–203 (2019).
